# The Feasibility Study of Megavoltage Computed Tomographic (MVCT) Image for Texture Feature Analysis

**DOI:** 10.3389/fonc.2018.00586

**Published:** 2018-12-05

**Authors:** Jiabing Gu, Jian Zhu, Qingtao Qiu, Yungang Wang, Tong Bai, Jinghao Duan, Yong Yin

**Affiliations:** ^1^School of Medicine and Life Sciences, University of Jinan-Shandong Academy of Medical Sciences, Jinan, China; ^2^Department of Radiation Oncology Physics and Technology, Shandong Cancer Hospital Affiliated to Shandong University, Shandong Academy of Medical Sciences, Jinan, China

**Keywords:** radiotherapy, helical tomotherpy, megavoltage computed tomotherapy, radiomics, feature analysis

## Abstract

**Purpose:** To determine whether radiomics texture features can be reproducibly obtained from megavoltage computed tomographic (MVCT) images acquired by Helical TomoTherapy (HT) with different imaging conditions.

**Methods:** For each of the 195 textures enrolled, the mean intrapatient difference, which is considered to be the benchmark for reproducibility, was calculated from the MVCT images of 22 patients with early-stage non-small-cell lung cancer. Test–retest MVCT images of an in-house designed phantom were acquired to determine the concordance correlation coefficient (CCC) for these 195 texture features. Features with high reproducibility (CCC > 0.9) in the phantom test–retest set were investigated for sensitivities to different imaging protocols, scatter levels, and motion frequencies using a wood phantom and *in-vitro* animal tissues.

**Results:** Of the 195 features, 165 (85%) features had CCC > 0.9. For the wood phantom, 124 features were reproducible in two kinds of scatter materials, and further investigations were performed on these features. For animal tissues, 108 features passed the criteria for reproducibility when one layer of scatter was covered, while 106 and 108 features of *in-vitro* liver and bone passed with two layers of scatter, respectively. Considering the effect of differing acquisition pitch (AcP), 97 features extracted from wood passed, while 103 and 59 features extracted from *in-vitro* liver and bone passed, respectively. Different reconstruction intervals (RI) had a small effect on the stability of the feature value. When AcP and RI were held consistent without motion, all 124 features calculated from wood passed, and a majority (122 of 124) of the features passed when imaging with a “fine” AcP with different RIs. However, only 55 and 40 features passed with motion frequencies of 20 and 25 beats per minute, respectively.

**Conclusion:** Motion frequency has a significant impact on MVCT texture features, and features from MVCT were more reproducibility in different scatter conditions than those from CBCT. Considering the effects of AcP and RI, the scanning protocols should be kept consistent when MVCT images are used for feature analysis. Some radiomics features from HT MVCT images are reproducible and could be used for creating clinical prediction models in the future.

## Introduction

Helical Tomotherapy (HT) has been widely used for intensity-modulated radiation therapy (IMRT) (1, 2). Compared with kilo-voltage cone beam computed tomotherapy (kV-CBCT) on a traditional external beam medical linear accelerator, megavoltage computed tomotherapy (MVCT) has a lower imaging dose (3) and larger scanning range. As a result, daily MVCT scans have been widely used for setup verification (4). However, these daily MVCT images contain not only setup information but also variations of the tumor and organs at risk. Therefore, to improve treatment outcomes, these daily MVCT images should be used to their full advantage to predict prognosis and complications and to guide treatment plan modification.

Studies have demonstrated that extracting texture features from computer tomography (CT) (5–7), contrast-enhanced CT (8, 9), or positron emission tomography (PET) (10–12) images using radiomics techniques may be used to predict cancer diagnosis (13), tumor hypoxia status (14), metastases (15), and irradiation-induced complications (16). Moreover, reproducible delta radiomics features extracted from CBCT can potentially be used for adaptive radiotherapy treatment decisions (17, 18). Therefore, texture features extracted from MVCT images may also have the potential to predict clinical outcomes.

Fave et al. (19) confirmed that some radiomics features from kV-CBCT are robust to noise and poor image quality when the imaging protocol is consistent. However, MVCT images are more noisy than CBCT images due, in part, to the less efficient detection of megavoltage X-rays relative to kilo-voltage X-rays (20). Compared to helical kilo-voltage CT (kV-CT), MVCT scans take longer and include more respiration cycles, which may result in more motion artifacts. Furthermore, the acquisition pitch (AcP) and reconstruction interval (RI) can be varied in the process of MVCT image acquisition. Therefore, the reproducibility of radiomics features extracted from MVCT images must be investigated to ensure that models built from these features can be consistently and reliably applied. To our knowledge, no studies have examined the robustness and reproducibility of texture features extracted from MVCT images.

The purpose of this study is to determine whether radiomics texture features can be reproducibly obtained from MVCT images and to investigate the impact of different imaging protocols, scatter, and motion frequency on the extracted features.

## Materials and Methods

**Figure 1:**
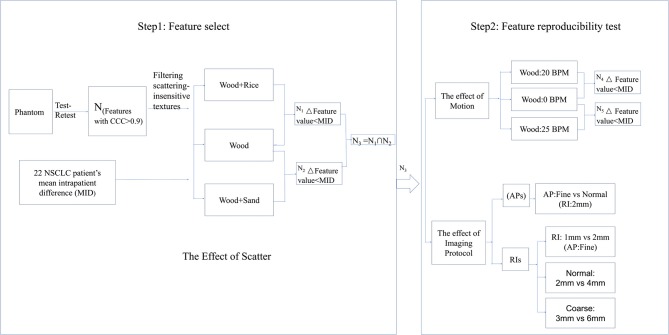
Flow chart of feature selection and the feature reproducibility tests.

This study consists of two steps: feature selection and feature reproducibility tests. The flow chart for these steps is shown in Figure 1. In the first step, the mean intrapatient difference (MID) of texture feature values was investigated as the benchmark for reproducibility. By referring to the benchmark, texture features with small delta values from phantom images with different artificial scatters (N_1_ and N_2_ in Figure 1) were selected. In the second step, the repeatability of the selected features (N_3_ in Figure 1) was verified using different motion frequencies and different imaging protocols.

### Patient MVCT Images

The prospective database consisted of MVCT images from 22 patients with early-stage non-small-cell lung cancer (NSCLC) who were treated with curatively intended radiotherapy using a HT system (Accuray ™ version 5.0, Madison, WI, USA) at Shandong Cancer Hospital affiliate to Shandong University. The MVCT images from the first and second fractions were analyzed to create a filtering criterion for evaluating the reproducibility of texture features. All patient MVCT images were acquired using a “fine” acquisition pitch (AcP) and a 1-mm reconstruction interval (RI). The gross tumor volume (GTV), which is defined as the primary tumor without lymph nodes, was recontoured from the MVCT images after rigid registration with treatment planning CT images using the Eclipse^TM^ Treatment Planning System (Varian Medical Systems, Inc., version 13.6, USA). Radiomic features were automatically extracted from the GTV of all MVCT images. Mathematical descriptions of the extracted 195 features are described. For the patient features, the MID was calculated with

(1) $$Mean\;intrapatient\;difference=\frac{\sum_{n=1}^{N_{pats}}\left|\left(\;x_{n,t}-x_{n,r}\right)\right|}{N_{pats}}$$

where *N*_*pats*_ is the number of patients, and *x*_*n, t*_ and *x*_*n, r*_ are the nth patient's first and second MVCT feature values, respectively. Because a phantom should change substantially less from scan-to-scan than a patient, we used the mean intrapatient difference as the benchmark for reproducibility in the phantom studies.

### Phantom Test–Retest MVCT Images

**Figure 2:**
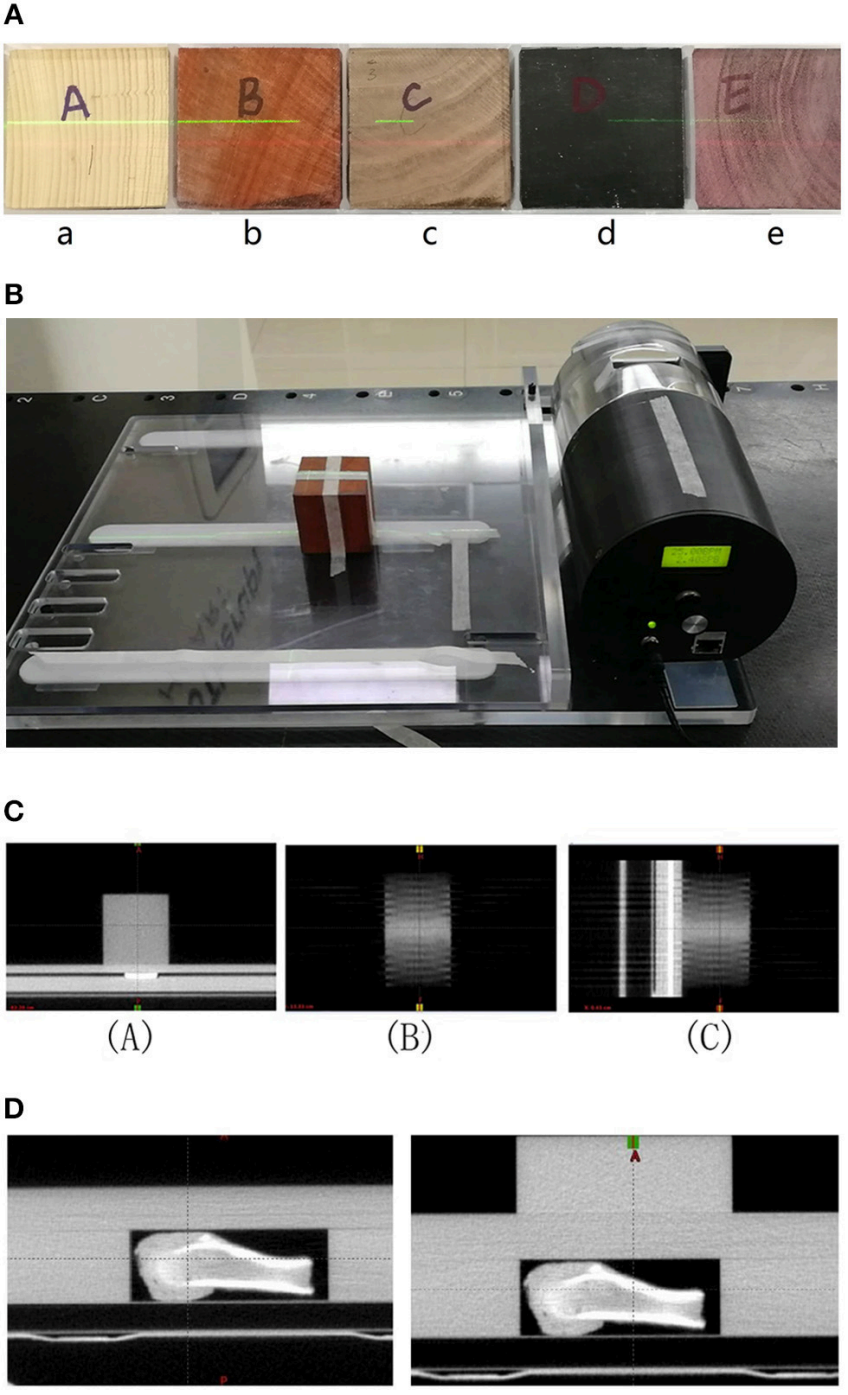
**(A)** Part of the radiomics phantom including 4 blocks of wood (a, b, c, and e) and one block of rubber (d). **(B)** The dynamic motion phantom with one block of wood. **(C)** Images of wood in motion at 20 BPMs [(A): transversal, (B): frontal, and (C): sagittal]. **(D)** Images of bone surrounded by different thicknesses of solid water.

Test–retest methods are often used in radiomics for eliminating unstable features. To facilitate test–retest methods, an in-house phantom was developed comprising 10 cartridges. Each 5 × 5 × 5 cm3 cartridge contains five kinds of wood, two kinds of sponge, and one kind of rubber. Figure 2A shows part of the radiomics phantom used in this study. Two sets of test and retest HT MVCT images were obtained for each of the ten blocks in the in-house texture phantom in a 15-min interval with AcP of “fine.” Images were reconstructed with a 512 × 512 grid size and 1-mm RI. To guarantee the region of interest (ROI) was in the same position for texture extraction, 10 ROIs were contoured on the test image for each of the ten materials and copied to the retest images after rigid registration.

For each of the 195 features, the concordance correlation coefficient (CCC) value was calculated using the test–retest image set. If average CCC value of 10 blocks was < 0.9, the features were considered to be non-repeatable and were excluded from the remaining analysis steps (21). The cutoff value of 0.9 was chosen according to the McBride criteria where a correlation of 0.9 reflects medium-consistency intensity. Therefore, this test removed features that were not reproducible in images obtained from a static phantom in a 15-min scanning interval with the same scanning options.

### Feature Extraction

Radiomic features were automatically extracted from the GTV of each patient image and the ROIs of phantom image using the open-source Imaging Biomarker EXplorer (IBEX) software (http://bit.ly/IBEX_MDAnderson) (22). Selected features included first-order descriptors from the intensity histogram (IH) and intensity direct (ID), second-order features describing spatial relationships in gray-level intensities from the co-occurrence matrix (COM), gray-level run-length matrix (GLRLM), and neighbor-intensity difference (NID), shape features describing the volume of the ROI convex hull calculated according to the 3D connectivity of adjacent voxels in binary masks, the gradient orient histogram (GoH) describing the intensity orientation to capture the prominent direction of intensity change. The detailed description of categories and radiomic features can be found in Supplementary Material.

Before calculating the feature functions, the images were rescaled to 8-bit images to reduce the effect of noise on the texture features and to prevent the generation of sparse matrices.

### Effect of Scatter

**Figure 3:**
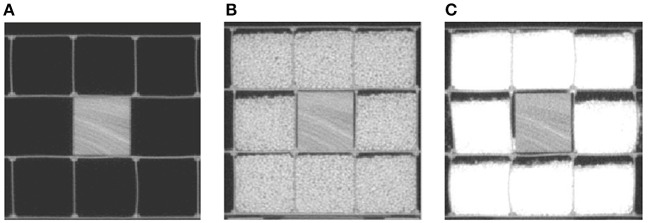
The MVCT images of wood surrounded by different kinds of scatter materials: **(A)** no scatter, **(B)** 5 cm of rice, and **(C)** 5 cm of sand.

The effect of scatter, generated by the different sizes of patients, on texture feature values is unknown. To determine whether the feature values were stable in different scatter, we first imaged wood, then wood surrounded by a 5-cm-thick layer of rice, and then wood surrounded by a 5-cm-thick layer of sand, as shown in Figure 3. We then imaged the animal tissues (liver, bone), the animal tissues with one layer of solid water, and the animal tissues with two layers of solid water, as shown in Figure 2D. The phantom and tissues were imaged with and without the scatter materials by HT MVCT with AcP of “fine” and RI of 1 mm.

The absolute differences in each of the features between the images with scatter materials and those with no scatter materials were calculated. The log of the ratio of the differences in phantom values to the MID was then calculated as the metric for this test:

(2) $$\begin{array}{l} {\text{log}}_{10}\left(\frac{phantom\;diff}{mean\;intrapatent\;diff}\right) \end{array}$$

When the phantom difference was less than the MID, the value was negative and passed. When the value was positive, the phantom difference was greater, and therefore, the feature failed. Considering that scatter cannot be avoided on MVCT images, only the features that passed were enrolled in the following analysis.

### Effect of AcPs and RIs

Different AcPs and RIs could impact adaptive dose calculation and registration. A smaller AcP value is beneficial for image registration but requires a long scan time (23). To determine the effects of AcPs and RIs, we imaged the wood texture phantom and animal tissues using the same MVCT imaging system under various scanning conditions. The MVCT images were obtained using six groups of scan settings. The groups consisted of three AcP options of “fine” (4 mm/rotation), “normal” (8 mm/rotation), and “coarse” (12 mm/rotation) multiplied by the two corresponding RIs (fine: 1 and 2 mm; normal: 2 and 4 mm; coarse: 3 and 6 mm). For each feature that passed the abovementioned tests, the absolute difference between the different AcPs and RIs were calculated, and those differences were compared to MID. If the difference between various imaged conditions was less than the MID, the features were considered to be reproducible between different scanning conditions.

### Effect of Motion

To analyze the effect of motion, the Quasar^®^ (Modus Medical Devices, London, ON) respiratory phantom carrying a 5 × 5 × 5 cm3 block of the wood texture phantom was utilized as shown in Figure 2B. A motion range of 2 cm with motion frequencies of 0, 20, and 25 beats per minute (BPM) was used with the dynamic motion phantom to determine whether features were reproducible under different motion conditions. The MVCT images were acquired using AcP of “fine” and RI of 1 mm. The images are shown in Figure 2C.

A 3D ROI was manually delineated on the MVCT images of the fixed wood block and then mapped to the MVCT images with different motion frequencies using rigid registration. Feature values were then calculated for each image set. The absolute difference between the texture value measured from images with and without motion was calculated and used as the phantom difference values in the numerator of Equation (2). A negative value implied that the phantom differences were less than the MID, and the feature passed. A positive value implied that the phantom differences were larger, and thus, the feature failed and was excluded from reproducible features.

## Results

### Phantom Test–Retest Results

**Figure 4:**
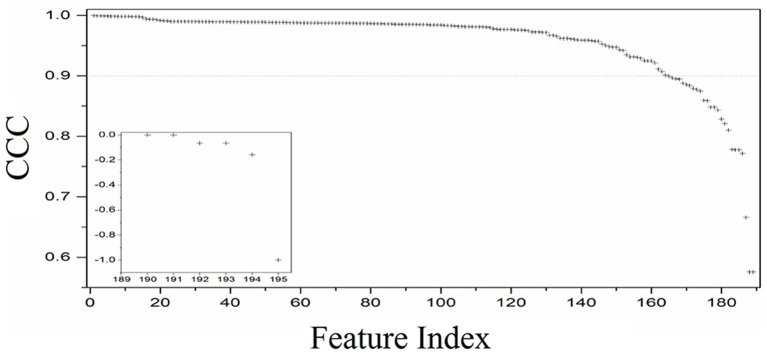
Distribution of the CCC values for the 195 texture features extracted from the in-house texture phantoms the test–retest data.

**Figure 5:**
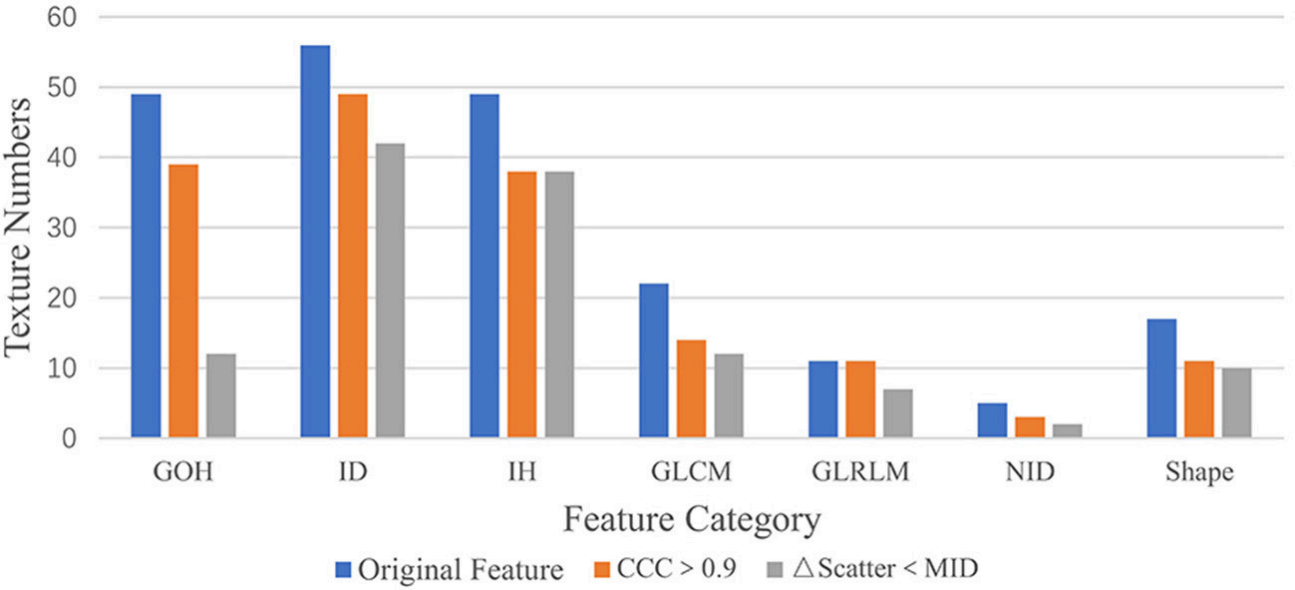
The texture numbers for the seven feature categories. An “Original Feature” is one of the 195 features extracted from 22 patient datasets, “Feature CCC > 0.9” is a feature with a CCC value >0.9, and “ΔFeature Scatter < MID” has a delta feature value less than the mean intrapatient difference for a wood block.

In the first part of this study, the CCC was used to evaluate reproducibility. Of the 195 texture features extracted from the phantom, 165 (N, 80%) had CCC > 0.9 (Figure 4). These included 39 features from GoH, 49 features from ID, 38 features from IH, 22 features from GLCM3, 11 features from GLRLM, 3 features from ND, and 11 shape features (Figure 5).

### Effect of Scatter

We examined whether the addition of scattering material will introduce changes to the texture features that were larger than the MID. For the wood, 136 (N_1_) of the 165 (82%) features were reproducible with rice scattering material, while 124 (N_2_ = N_3_, 75%) features were reproducible with sand scattering material.

For the liver, 108 of 124 (87%) features were reproducible with one layer of solid water, and 106 (85%) features were reproducible with two layers of solid water. For the bone, the same 108 of 124 (87%) features were reproducible under different scattering conditions. The results from the comparison of features calculated with different scattering materials are shown in Figure 5.

### Effect of ACPS and RIs

In this part of the study, we examined whether changing the AcP resulted in changes in texture features that were larger than the MID. For the wood, all 124 features with CCC > 0.9 passed the comparison in the same imaging AcP and RI. However, with different AcP settings (fine, normal, coarse), only 97 of the 124 (78%) features passed. For animal liver and bone, 103 (83%) and 59 (47%) features passed with different AcP settings, respectively. Results from the comparison of features calculated with different AcPs are shown in Figure 7.

**Figure 6:**
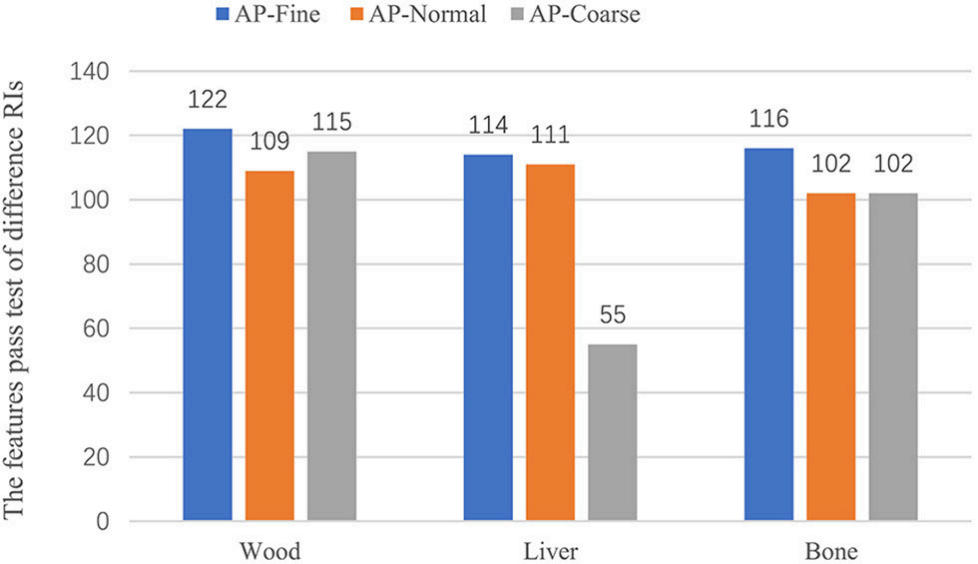
Result of the RI tests for wood and tissue. The test included three AcPs (fine, normal, and coarse) with two RIs for each AcP.

**Figure 7:**
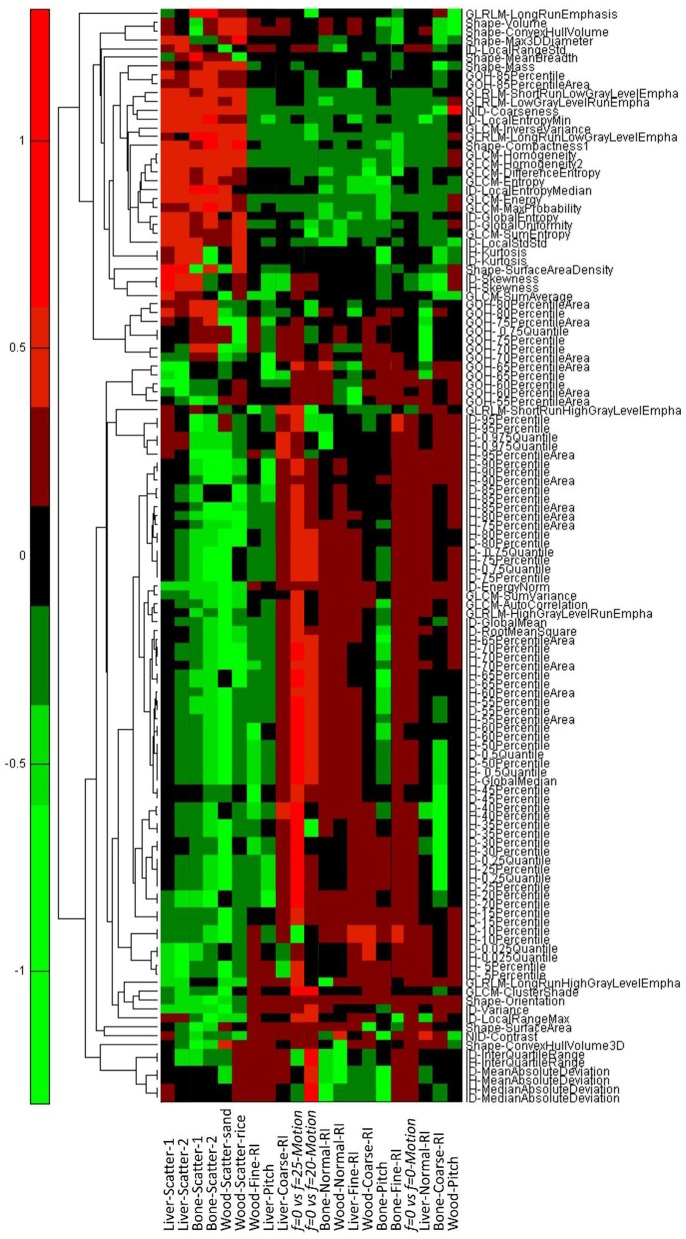
The results for the scatter, AcP, RI, and motion tests. Negative values imply the difference in the phantom measurements is less than the mean intrapatient difference and thus a “pass.” Positive values imply that the phantom difference was larger than the mean intrapatient difference, and the feature failed the test. Scatter1 indicates tissues surrounded with one layer of solid water, and scatter2 indicates tissues surrounded with two layers of solid water.

We also investigated the effect of different RIs on the stability of the texture features. Figure 6 shows the features, which were reproducible under imaging with different AcPs, imaged with different RIs. For a wood, imaged twice at an AcP of “fine” and RI of 1 mm, all 124 features passed the test; however, with an AcP of “fine” with different RIs (1 mm, 2 mm), ShortRunLowGrayLevelEmpha feature in the GLRLM category failed. Thus, features were more reproducible with an AcP of “fine.” Results from the comparison of features calculated with different RIs are shown in Figure 7.

### Effect of Motion

In the fourth part of this study, we examined whether motion frequencies would produce changes in the texture features that were larger than the MID. Without motion, all of the 124 features that passed the AcP and RI study were reproducible, while only 55 (N_4_, 44%) and 40 (N_5_, 32%) features passed when the frequency of motion was 20 and 25 BPM, respectively. There were 33 features that passed under both motion frequencies. Results from the comparison of features calculated with different frequencies of motion are shown in Figure 7.

### Reproducibility of Clinically Significant Features

In the last part of the study, 23 features that were significantly related to clinical factors based on CT images were selected (Table 1), and their robustness on MVCT images was evaluated. In this test, if the delta feature values for various motion frequencies were greater than the MID but in the range of 0.1, the features were still considered to be stable because the motion range and frequency in this study was larger than that for most patients in clinical practice. Compared with scatter, different AcPs and motion frequencies had more effect on the stability of feature values. For wood, nine clinically significant MVCT features, which have been used in previous texture feature studies on CT images of NSCLC or liver tumor diagnosis, were insensitive to motion. Only 6 of the 23 (26%) features from four categories were reproduceable in different AcPs.

**Table 1:** Reproducibilities of clinically significant features.

**Features**	**Clinical relative factor**	**AcP**			**Scatter**			**Motion_wood**		
		**Wood**	**Bone**	**Liver**	**Wood**	**Liver**	**Bone**	**0BPM**	**20BPM**	**25BPM**
IH_Kurtosis (6, 24, 25)	Predictor of overall survival in HN and NSCLC patients	×	×	✓	✓✓	✓✓	✓✓	✓	×	✓
IH_0.75Quantile (26)	Correlation of radiation pneumonitis development	✓	✓	✓	✓✓	✓✓	✓✓	✓	×	×
IH_Skewness (24, 25)	Predictor of overall survival in HN and NSCLC patients	×	✓	✓	✓✓	✓✓	✓✓	✓	×	×
GLCM_AutoCorrelation (7)	Decoding tumor phenotype	✓	✓	✓	✓✓	✓✓	✓✓	✓	×	×
GLCM_ClusterShade (7, 23)	Predicts distant metastasis in lung adenocarcinoma, decoding tumor phenotype	✓	✓	✓	✓✓	✓✓	✓✓	✓	×	×
GLCM_DiffEntropy (6, 7, 27)	Predictor for malignant pulmonary nodules, decoding tumor phenotype	×	✓	✓	✓✓	✓✓	✓✓	✓	✓	✓
GLCM_Energy (7, 27, 28)	Predictor for malignant pulmonary nodules and hepatocellular carcinoma, decoding tumor phenotype,	×	✓	✓	✓✓	✓✓	✓✓	✓	✓	✓
GLCM_Entropy (6, 24, 7, 27, 29)	Predictor of overall survival in HN, NSCLC Patients and malignant pulmonary nodules, decoding tumor phenotype	×	×	✓	✓✓	✓✓	✓✓	✓	✓	✓
GLCM_ Homogeneity (27, 28, 29, 30)	Predictor local control and survival in hepatocellular carcinoma patients, assessment of structural changes in parotid glands	×	×	✓	✓✓	✓✓	×✓	✓	✓	×
GLCM_ Homogeneity2 (7)	Decoding tumor phenotype	×	×	✓	✓✓	✓✓	×✓	✓	✓	×
GLCM_ InverseVariance (7)	Decoding tumor phenotype	×	✓	✓	✓✓	×✓	✓×	✓	✓	✓
GLCM_ MaxProbability (7, 27)	Decoding tumor phenotype, prediction model for malignant pulmonary nodules	×	✓	✓	✓✓	✓✓	✓✓	✓	✓	✓
GLCM_ SumAverage (6, 26, 7)	Correlation of radiation pneumonitis development, decoding tumor phenotype	✓	×	✓	✓✓	✓✓	✓✓	✓	×	×
GLCM_ SumEntropy (6, 26, 27)	Correlation of radiation pneumonitis development	×	✓	✓	✓✓	✓✓	✓✓	✓	✓	✓
GLCM_ SumVariance (6, 7)	Decoding tumor phenotype	✓	✓	✓	✓✓	✓✓	✓✓	✓	×	×
GLRM_ HighGrayLevelRunEmpha (7)	Decoding tumor phenotype	✓	✓	✓	✓✓	✓✓	✓✓	✓	×	×
GLRM_ LongRunEmphasis (24, 7, 28)	Prediction of overall survival in HN Patients and hepatocellular carcinoma patients	×	×	✓	✓✓	✓✓	× ×	✓	✓	×
GLRM_LongRunHighGrayLevelEmpha (7)	Decoding tumor phenotype	✓	×	✓	✓✓	✓✓	× ×	✓	×	×
GLRM_LongRunLowGrayLevelEmpha (7)	Decoding tumor phenotype	×	×	✓	✓✓	✓✓	× ×	✓	✓	✓
GLRM_ LowGrayLevelRunEmpha (7)	Decoding tumor phenotype	×	×	✓	✓✓	✓✓	✓✓	✓	✓	✓
GLRM_ShortRunHighGrayLevelEmpha (7)	Decoding tumor phenotype	✓	✓	×	✓✓	×✓	✓✓	✓	×	×
GLRM_ShortRunLowGrayLevelEmpha (7)	Decoding tumor phenotype	×	×	✓	✓✓	✓✓	×✓	✓	✓	✓
ID_0.75Quantile (26)	Correlation of radiation pneumonitis development	✓	✓	✓	✓✓	✓✓	✓✓	✓	×	×

“*×” indicates a “fail,” meaning the feature is not reproducible under such conditions. “✓”indicates a “pass,” meaning the feature can be reproducible under such conditions. “✓✓” indicates the results passed in two scattering conditions*.

These results indicate that most of those features were insensitive to scatter. However, in practical clinical practice, there may not be large scattering differences in the patient's MVCT imaging during the radiotherapy course. Moreover, this result was expected because the scattering in MVCT imaging is mainly Compton scattering, which is independent of the atomic number of the imaging object, while the scattering in kV-CT imaging is mainly due to the photoelectric effect, which is dependent on the atomic number of the imaging object and, thus, more sensitive to scatter.

## Discussion

Texture features extracted from kV-CT images can be used to predict distant metastases in oropharyngeal carcinoma (31), assess radiation-induced lung injury after stereotactic body radiotherapy (32), and act as imaging correlates for tumor hypoxia and angiogenesis for NSCLC (8). In this study, we found some texture features were reproducible in MVCT images. However, MVCT has poorer image quality (spatial resolution) than kV-CT and less functional information than positron emission tomography (PET). Several studies have reported different solutions to improve the MVCT image quality for clinical applications (33). A discriminative feature representation method, which was modified by a block matching and 3D filtering (BM3D) algorithm, has been shown to yield significant improvement in soft tissue contractions. Accuray^TM^ Incorporated has developed a new version of the Radixact system software to reduce noise and provide better soft-tissue contrast with the same low imaging dose. With these changes, differences in anatomy will be more apparent, allowing more confident use of MVCT images for delineating ROIs and for texture analysis. In addition to the existing improvements, it may also be useful to perform texture enhancements before texture feature analysis.

From our results, some MVCT features are reproducible under certain circumstances. Several of these reproducible features have been found to be useful in studies using kV-CT or PET-CT images (Table 1). For example, the reproducibility of IH_Kurtosis in MVCT images can be used to predict the overall survival in both head and neck cancer and NSCLC patients, while GLCM_Entropy can be used to predict the overall survival in head and neck cancer. The links shown in Table 1 are encouraging, suggesting potential that reproducible features can be used to build models for predicting prognosis.

In previous studies, Fave et al. explored the reproducibility of CBCT features to differences in imaging protocol, levels of scatter, and amounts of motion using phantoms. During a radiotherapy course, MVCT images are acquired every fraction, and the scan range can be selected very conveniently. The reproducibility of MVCT features should be assessed before MVCT images are used for feature analysis. Considering the characteristics of MVCT, we explored the effects of RI and AcP on features for the MVCT image protocol in addition to differences in imaging protocol, levels of scatter, and amounts of motion.

### Phantom Test-Retest

Radiomic features that qualify as potential imaging biomarkers must be robust, that is, insensitive to data acquisition protocols, scatter, and motion frequencies. Test–retest methods are often used to eliminate unstable features (15–20). To minimize the possibility of false positives, conservative cutoffs were deliberately adopted in this study. Despite such strict reproducibility standards (CCC > 0.9), a relatively high pass rate occurred for features obtained from MVCT images. Approximately 84.6% of the features initially considered passed this qualifying test, and those features covered every texture category. The large number and wide variety of features that passed offer preliminary support for the possibility of texture analysis in MVCT images.

### Effect of Scatter

Scattering is inevitable during MVCT image acquisition; therefore, feature values that changed more than the MID were removed. Studies on reproducible features obtained from CBCT found that almost all features changed substantially when scatter material was added around the phantom (19). However, for wood, our results found that 75.15% of features with CCC > 0.9 changed less than the MID, which indicated that MVCT features are more reproducible in various scatter conditions than CBCT features. To confirm this result, 124 features from animal tissues were analyzed, and most of those features were still considered reproducible. In patient clinical practice, the change in the amount of scatter would likely be substantially less than that on the phantom. Thus, the features obtained from tissue images that did not pass this evaluation may still be used for feature analysis in clinical analysis if the change in scatter is not significant. The scatter materials surrounding the tissues were uniform in this study, but scatter materials surrounding the tumor will not be uniform *in-vivo*. Therefore, compared to clinical practice, this study might suggest a reduced effect of scattering material with a subsequently larger number of reproducible features.

### Effect of AcP and RI

Fave et al. investigated the reproducibility of CBCT features with different imaging protocols, levels of scatter, and amounts of motion. In addition to these factors, our study also investigated the effects of RI and AcP on features for the MVCT imaging protocol. At the TomoTherapy Operator Station (Version 5.0, Accuray, Sunnyvale, CA), there are options for three AcP values, and the AcP is directly connected to the couch speed, scan duration, and patient absorption dose (34). Studies on the impact of AcP on image quality have argued that there is no noticeable difference in the image quality among different AcPs (35) or the differences could be ignored (36). Our study found that some features were reproducible with imaging under different AcPs, and those reproducible features covered six categories. The reproducible features could be used for feature analysis without considering the effect of AcPs, which might be convenient for retrospective MVCT feature studies. Moreover, this broad spectrum of reproducible features is helpful because features from different categories can provide independent information about an image and combinations of these categories can provide a more complete picture.

To consider the effect of thickness, we compared scans with 2-mm RI and AcPs of “fine” and “normal.” When imaged with the same AcP and thickness values, all 124 features extracted from wood with CCC > 0.9 passed the comparison, which indicated that features were reproducible with the same AcPs. However, some feature values obtained from images obtained at the same thickness and different AcPs were not reproducible. The AcPs have different effects on features in different materials, and the most obvious effect was in the bone. The results indicate that the scanning protocol should be kept consistent when analyzing radiomics features extracted from MVCT images. The results also revealed that more features were reproducible if they were obtained from wood and liver than if they were obtained from bone. The density of bone is relatively higher than that of other tissues. Furthermore, bone has a larger range of density variation (Figure 2D), which might be more sensitive to irradiation during imaging.

The thickness of the reconstruction does not affect the scan time and the patient absorption dose; therefore, we recommend using the smallest available RI value in clinical practice. Considering the trade-off between IGRT and adaptive radiotherapy, Zhu et al. (37) recommended an AcP of “normal” with 2-mm RI in daily practice. For an AcP of “normal,” we found that some features were significantly affected by the slice intervals. Therefore, we recommend using the same slice interval in clinical practice, which may reduce the effect of slice intervals on MVCT feature reproducibility.

### Effect of Motion

Motion of an imaging object will increase the uncertainty of texture feature values. For MVCT, we assumed that the effect of motion might be larger than that in conventional kV-CT because of the longer imaging time, which contains more motion periods. In particular, for chest and abdomen tumors, the effect of respiratory motion on texture values is unavoidable during HT MVCT radiotherapy. Most feature values change with increasing motion of the tumor. Fave et al. recommend a threshold of at most 10 mm and potentially as low as 5 mm for future studies because the majority of patients with NSCLC had tumor motion that was < 5 mm and only 10% had motion that was >10 mm (25). However, in our study, to filter out texture values that were less affected by movement, a larger range of motion was utilized. At the same time, to identify more reproducible texture features, the effect of different motion frequencies was also investigated. The larger range of motion may have eliminated some potential textures that can be used in the future but should identify more reproducible values for clinical applications.

Some MVCT features were reproducible under the harsh motion conditions of our tests. The reproducible features were found in seven categories, providing a large amount of image information. These reproducible MVCT features might be used for chest or abdomen tumors where it is important to ignore the effect of motion.

## Conclusion

Motion frequency has a significant impact on MVCT features, and the effects from faster motion frequencies are more obvious. Texture features from MVCT were more reproducible in various scatter conditions than those from CBCT. Some of the reproducible radiomics features for MVCT images could be used for creating clinical prediction models in the future. Considering the significant effect of AcP and RI on the texture features, the scanning protocol should be kept consistent when MVCT images are used for feature analysis.

## Ethics Statement

This work was approved by the ethics committee of Shandong Cancer Hospital Affiliated to Shandong University. The need for informed consent was waived by the Medical Ethics Committee because the study was an observational, retrospective study using a database from which the patients' identifying information had been removed.

## Author Contributions

JG and JZ designed the experiment, analyzed the experimental raw data, and was a major contributor in writing the manuscript. TB and YW executed the experiment process, recorded the data and revised the manuscript for important intellectual contents. QQ and JD checked the experimental raw data. YY made substantial contributions to conception and design the whole experiment. All authors read and approved the final manuscript.

### Conflict of Interest Statement

The authors declare that the research was conducted in the absence of any commercial or financial relationships that could be construed as a potential conflict of interest.
