# Supplementary material for: The Feasibility Study of Megavoltage Computed Tomographic (MVCT) Image for Texture Feature Analysis
Source: Front Oncol. 2018 Dec 5;8:586. doi: 10.3389/fonc.2018.00586 (PMC6290333; doi:10.3389/fonc.2018.00586)
Supplement: Supplementary file 1 [file Table_2.DOCX]

Supplementary Material

The Feasibility Study of Megavoltage Computed Tomographic (MVCT) Image for Texture Feature Analysis

Jiabing Gu*, Jian Zhu, Qingtao Qiu, Yungang Wang, Tong Bai, Jinghao Duan, Yong Yin

Correspondence: Yong Yin: yinyongsd@126.com

Jian Zhu: zhujian.cn@163.com

**Radiomics Features**

The supplementary information contains mathematical definitions of extracted radiomic features by using IBEX. We list the reproducibility features in MVCT images, and describe some of the features commonly used in the clinical research.

Group 1. First odder statistics

Group 2. Textural features

Group 3. Shape and size based features

Group 1. First order statistics

First-order descriptors from the intensity histogram (IH), intensity direct (ID) and gradient orient histogram (GoH)are describing the distribution of voxel intensities within the MVCT images through commonly used and basic metrics. Intensity histogram is to compute histogram from image inside the binary mask. Intensity direct is to preprocess the binary mask for the features derived directly from the image intensity. The following first order statistics were extracted:

**Intensity Histogram**

1.Kurtosis: Measure the peakedness of the occurence probability values in the histogram.

$$kurtosis=\frac{\frac{1}{N_{p}}\sum_{i=1}^{N_{p}} {(\mathbf{X}\left( i \right)-\bar{X})}^{4}}{{(\frac{1}{N_{p}}\sqrt{\sum_{i=1}^{N_{p}} {(\mathbf{X}\left( i \right)-\bar{X})}^{2}})}^{2}}$$

2. Skewness: Measure the asymmetry of the occurence probability values in the histogram.

$$skewness=\frac{\frac{1}{N_{p}}\sum_{i=1}^{N_{p}} {(\mathbf{X}\left( i \right)-\bar{X})}^{3}}{{(\sqrt{\frac{1}{N_{p}}\sum_{i=1}^{N_{p}} {(\mathbf{X}\left( i \right)-\bar{X})}^{2}})}^{3}}$$

3.nth percentile: The nth percentile of **X**

4. PercentileArea: Percentiles of values in the accumulative histogram.

5. Quantile: Quantiles of the occurence probability values in the histogram

6. MeanAbsoluteDeviation: The mean absolute deviation of the occurence probability values in the histogram.

$$MAD=\frac{1}{N_{p}}\sum_{i=1}^{N_{p}} \left| \mathbf{X}\left( i \right)-\bar{X} \right|$$

7. MedianAbsoluteDeviation: The median absolute deviation of the occurence probability values in the histogram.

8. InterQuartileRange: The interquartile range of the occurence probability values in the histogram. $interquartile range=P_{75}-P_{25}$. Here, $P_{75}=75th percentile$ and $P_{25}=25th percentile$.

9. Range: Measure the range (MaxValue-MinValue) of the occurence probability values in the histogram.

**Intensity Direct**

1. Kurtosis: $kurtosis=\frac{\frac{1}{N_{p}}\sum_{i=1}^{N_{p}} {(\mathbf{X}\left( i \right)-\bar{X})}^{4}}{{(\frac{1}{N_{p}}\sqrt{\sum_{i=1}^{N_{p}} {(\mathbf{X}\left( i \right)-\bar{X})}^{2}})}^{2}}$

2.Skewness: $skewness=\frac{\frac{1}{N_{p}}\sum_{i=1}^{N_{p}} {(\mathbf{X}\left( i \right)-\bar{X})}^{3}}{{(\sqrt{\frac{1}{N_{p}}\sum_{i=1}^{N_{p}} {(\mathbf{X}\left( i \right)-\bar{X})}^{2}})}^{3}}$

3. Energy:$energy=\sum_{i=1}^{N_{p}} {(\mathbf{X}\left( i \right)+c)}^{2}$

4. GlobalUniformity: The intensity uniformity among all the voxels.

5. LocalEntropyStd: Step1, at each voxel, compute entropy in its neighborhood region. And then, compute the standard deviation among all the voxel's entropy caculated from step1.

6. Percentile: Percentiles of the intensity values among all the voxels.

7. GlobalMedian: The intensity median among all the voxels.

8. Quantile: Quantiles of the intensity values among all the voxels.

9. GlobalMean: The intensity mean among all the voxels.

10. RootMeanSquare: $RMS=\sqrt{\frac{\sum_{i}^{N} \mathbf{X}{(i)}^{2}}{N}}$

11. GlobalEntropy: The intensity entropy among all the voxels.

12. LocalRangeStd: Stpe1, at each voxel, compute range value (MaxValue-MinValue) in its neighborhood region. Then, compute the standard deviation among all the voxel's range value caculated from step1.

13.LocalEntropyMedian: Step1, at each voxel, compute entropy in its neighborhood region.Then, compute the median among all the voxel's entropy caculated from step1.

14.LocalEntropyMean: Step1, at each voxel, compute entropy in its neighborhood region. Then, compute the mean among all the voxel's entropy caculated from step1.

15.LocalStdStd: Step1, at each voxel, compute standard deviation in its neighborhood region. Then, compute the standard deviation all the voxel's standard deviation value caculated from Step 1.

16.LocalRangeMean: Stpe1, at each voxel, compute range value (MaxValue-MinValue) in its neighborhood region. Then, compute the mean among all the voxel's range value caculated from step 1.

17. MeanAbsoluteDeviation: The mean absolute deviation of the intensity values among all the voxels.

18. LocalRangeMax: Step1, at each voxel, compute range value (MaxValue-MinValue) in its neighborhood region. Then, compute the median among all the voxel's range value caculated from step1.

19. InterQuartileRange: The interquartile range of the intensity values among all the voxels.

20. GlobalStd: The intensity standard deviation among all the voxels.

21. Variance:

$$variance=\frac{1}{N_{p}}\sum_{i=1}^{N_{p}} {(\mathbf{X}\left( i \right)-\bar{X})}^{2}$$

22. GlobalMax: The intensity maximum among all the voxels.

23. Range: The intensity range (MaxValue-MinValue) among all the voxels.

**Gradient Orient Histogram (GoH)**

1. Kurtosis: Measure the peakedness of the occurrence probability values in the histogram.

$$kurtosis=\frac{\frac{1}{N_{p}}\sum_{i=1}^{N_{p}} {(\mathbf{X}\left( i \right)-\bar{X})}^{4}}{{(\frac{1}{N_{p}}\sqrt{\sum_{i=1}^{N_{p}} {(\mathbf{X}\left( i \right)-\bar{X})}^{2}})}^{2}}$$

2. Percentile: Percentiles of the occurence probability values in the histogram.

3. PercentileArea: Percentiles of values in the accumulative histogram.

4. Quantile: Quantiles of the occurence probability values in the histogram.

5. MeanAbsoluteDeviation: The mean absolute deviation of the occurence probability values in the histogram.

6. InterQuartileRange: The interquartile range of the occurence probability values in the histogram.

7. MedianAbsoluteDeviation: The median absolute deviation of the occurence probability values in the histogram.

8. Range: Measure the range (MaxValue-MinValue) of the occurence probability values in the histogram.

9.Skewness: Measure the asymmetry of the occurence probability values in the histogram.

$$skewness=\frac{\frac{1}{N_{p}}\sum_{i=1}^{N_{p}} {(\mathbf{X}\left( i \right)-\bar{X})}^{3}}{{(\sqrt{\frac{1}{N_{p}}\sum_{i=1}^{N_{p}} {(\mathbf{X}\left( i \right)-\bar{X})}^{2}})}^{3}}$$

Group 2. Textural features

This group provide the information regarding the relative position of the various gray levels of the MVCT images. The second order features which describes spatial relationships in gray level intensities from the gray level co-occurrence matrix (COM), gray level run length matrix (GLRLM) and neighbor intensity difference3(NID) were calculated in this group.

**Gray Level Co-occurrence Matrix**

For a Gray Level Co-occurrence Matrix with size N_g_ x N_g_, it describes the second-order joint probability function of an image region and is defined as $\mathbf{P}\left( \boldsymbol{i,j} | \boldsymbol{\delta,\theta} \right)$. And, the ${(i,j)}^{th}$ element of this matrix represents the number of times the combination of levels $i$ and $j$ occur in two pixels in the image, that are separated by a distance of $\delta$ pixels along angle $\theta$. Let:

- $\epsilon$ be an arbitrarily small positive number ($\approx2.2\times{10}^{-16}$).
- $\mathbf{P}\left( i,j \right)$ be the co-occurence matrix for an arbitrary $\delta$ and $\theta$.
- $p\left( i,j \right)$ be the normalized co-occurence matrix and equal to $\frac{\mathbf{P}\left( i,j \right)}{\sum\mathbf{P}\left( i,j \right)}$.
- N_g_ be the number of discrete gray level within image.
- $p_{x}\left( i \right)=\sum_{j=1}^{N_{g}} \mathbf{P}\left( i,j \right)$ and $p_{y}\left( i \right)=\sum_{i=1}^{N_{g}} \mathbf{P}\left( i,j \right)$ be the marginal row and column probabilities, respectively.
- $\mu_{x}=\sum_{i=1}^{N_{g}} p_{x}\left( i \right)i$ and $\mu_{y}=\sum_{j=1}^{N_{g}} p_{y}\left( j \right)j$ be the mean gray level intensity of $p_{x}$ and $p_{y}$, respectively.
- $\sigma_{x}$ and $\sigma_{y}$ be the standard deviation of $p_{x}$ and $p_{y}$, respectively.
- $p_{x+y}\left( k \right)=\sum_{i=1}^{N_{g}} \sum_{j=1}^{N_{g}} p\left( i,j \right)$, where $i+j=k$, and $k=2, 3, \ldots, 2N_{g}$.
- $p_{x-y}\left( k \right)=\sum_{i=1}^{N_{g}} \sum_{j=1}^{N_{g}} p\left( i,j \right)$, where $\left| i-j \right|=k$, and $k=0, 1, \ldots, N_{g}$.
- $HX=-\sum_{i=1}^{N_{g}} p_{x}\left( i \right){log}_{2}(p_{x}\left( i \right)+\varepsilon)$ be the entropy of $p_{x}$.
- $HY=-\sum_{j=1}^{N_{g}} p_{y}\left( i \right){log}_{2}(p_{y}\left( j \right)+\varepsilon)$ be the entropy of $p_{y}$.
- $HXY=-\sum_{i=1}^{N_{g}} \sum_{j=1}^{N_{g}} p\left( i,j \right){log}_{2}(p(i,j)+\varepsilon)$ be the entropy of $p\left( i,j \right)$.
- $HXY1=-\sum_{i=1}^{N_{g}} \sum_{j=1}^{N_{g}} p\left( i,j \right){log}_{2}(p_{x}\left( i \right)p_{y}\left( j \right)+\epsilon)$.
- $HXY2=-\sum_{i=1}^{N_{g}} \sum_{j=1}^{N_{g}} p_{x}\left( i \right)p_{y}\left( j \right){log}_{2}(p_{x}\left( i \right)p_{y}\left( j \right)+\epsilon)$.

1. Autocorrelation:

$$Autocorrelation=\sum_{i=1}^{N_{g}} \sum_{j=1}^{N_{g}} \mathbf{p}\left( i,j \right)ij$$

2. ClusterProminence:

$$Cluster Prominence=\sum_{i=1}^{N_{g}} \sum_{j=1}^{N_{g}} \left( i+j-\mu_{x}-\mu_{y} \right)^{4}\mathbf{p}(i,j)$$

3. ClusterShade:

$$Cluster Shade=\sum_{i=1}^{N_{g}} \sum_{j=1}^{N_{g}} \left( i+j-\mu_{x}-\mu_{y} \right)^{3}\mathbf{p}(i,j)$$

4. Cluster Tendency:

$$Cluster Tendency=\sum_{i=1}^{N_{g}} \sum_{j=1}^{N_{g}} \left( i+j-\mu_{x}-\mu_{y} \right)^{2}\mathbf{p}(i,j)$$

5. Energy:

$$Energy=\sum_{i=1}^{N_{g}} \sum_{j=1}^{N_{g}} \left[ \mathbf{p}\left( i,j \right) \right]^{2}$$

6. Entropy:

$$Entropy=-\sum_{i=1}^{N_{g}} \mathbf{p}\left( i,j \right){log}_{2}[\mathbf{p}(i,j)]$$

7. Correlation:

$$correlation=\frac{\sum_{i=1}^{N_{g}} \sum_{j=1}^{N_{g}} p\left( i,j \right)ij-\mu_{x}\mu_{y}}{\sigma_{x}\left( i \right)\sigma_{y}\left( j \right)}$$

8. Contrast:

$$contrast=\sum_{i=1}^{N_{g}} \sum_{j=1}^{N_{g}} \left( i-j \right)^{2}\mathbf{p}\left( i,j \right)$$

9. Dissimilarity:

$$\mathrm{Dissimilarity}=\sum_{i=1}^{N_{g}} \sum_{j=1}^{N_{g}} \left| i-j \right|\mathbf{p}(i,j)$$

10.DifferenceEntropy:

$$DifferenceEntropy=\sum_{k=0}^{N_{g}-1} \mathbf{p}_{x-y}\left( i \right){log}_{2}\left( \mathbf{p}_{x-y}\left( i \right)+\epsilon\right)$$

11. Homogeneity:

$$\mathrm{Homogeneity}=\sum_{i=1}^{N_{g}} \sum_{j=1}^{N_{g}} \frac{\mathbf{p}(i,j)}{1+\left| i-j \right|}$$

|  |
| --- |
| 12.Homogeneity2: $Homogeneity2=\sum_{i=1}^{N_{g}} \sum_{j=1}^{N_{g}} \frac{\mathbf{p}(i,j)}{1+\left\vert i-j \right\vert^{2}}$ |
| 13.MaxProbability: $Maximum Probability=max\left( p\left( i,j \right) \right)$ 14.InverseVariance: $Inverse Variance=\sum_{i=1}^{N_{g}} \sum_{j=1}^{N_{g}} \frac{\mathbf{p}\left( i,j \right)}{\left\vert i-j \right\vert^{2}} , i\neq j$ 15. InverseDiffMomentNorm(IDMN): $IDMN=\sum_{i=1}^{N_{g}} \sum_{j=1}^{N_{g}} \frac{\mathbf{p}\left( i,j \right)}{1+\left( \frac{\left\vert i-j \right\vert^{2}}{N_{g}^{2}} \right)}$ 16.InformationMeasureCorr1: $IMC1=\frac{HXY-HXY1}{max\{HX,HY\}}$ 17.InformationMeasureCorr2: $IMC2=\sqrt{1-e^{-2(HXY2-HXY)}}$ 18.SumAverage: $sum average=\sum_{k=2}^{2N_{g}} \mathbf{p}_{x+y}(i)i$ 19.SumEntropy (SE): $SE=\sum_{k=2}^{2N_{g}} \mathbf{p}_{x+y}(i){log}_{2}\left( \mathbf{p}_{x+y}\left( i \right)+\epsilon\right)$ 20.SumVariance: $sum variance=\sum_{i=2}^{2N_{g}} \left( i-SE \right)^{2}\mathbf{p}_{x+y}(i)$ 21. Variance: $varian=\sum_{i=1}^{N_{g}} \sum_{j=1}^{N_{g}} \left( 1-u \right)^{2}\mathbf{p}(i.j)$ |

**Gray Level Run Length Matrix Features**

Gray Level Run Length Matrix quantifies gray level runs which is defined as the length in number of pixels, of consecutive pixels that have the same gray level value. In a gray level run length matrix *p*(i,j│θ), the (i,j)th element describes the number of runs with gray level i and length j occur in the image (ROI) along angle θ.

Let:

- $N_{g}$ be the number of discreet intensity values in the image.
- $N_{r}$ be the number of discreet run lengths in the image.
- $N_{p}$ be the number of voxels in the image.
- $N_{z}(\theta)$ be the number of runs in the image along angle $\theta$, which is equal to $\sum_{i=1}^{N_{g}} \sum_{j=1}^{N_{r}} \mathbf{P}\left( i,j | \theta\right)$ and $1<N_{z}(\theta)\leq N_{p}$.
- $\mathbf{P}\left( i,j | \theta\right)$ be the run length matrix for an arbitrary direction $\theta$.
- $p\left( i,j | \theta\right)=\frac{\mathbf{P}\left( i,j | \theta\right)}{N_{z}(\theta)}$ be the normalized run length matrix.

1.GrayLevelNonuniformity (GLN):

$$GLN=\frac{\sum_{i=1}^{N_{g}} ({\sum_{j=1}^{N_{r}} \mathbf{P}\left( i,j | \theta\right))}^{2}}{N_{z}(\theta)}$$

2. LowGrayLevelRunEmpha (LGLRE):

$$LGLRE=\frac{\sum_{i=1}^{N_{g}} \sum_{j=1}^{N_{r}} \frac{\mathbf{P}\left( i,j | \theta\right)}{i^{2}}}{N_{z}(\theta)}$$

3. LongRunLowGrayLevelEmpha (LRLGLE):

$$LRLGLE=\frac{\sum_{i=1}^{N_{g}} \sum_{j=1}^{N_{r}} \frac{\mathbf{P}\left( i,j | \theta\right)j^{2}}{i^{2}}}{N_{z}(\theta)}$$

4. LongRunHighGrayLevelEmpha (LRHGLE):

$$LRHGLE=\frac{\sum_{i=1}^{N_{g}} \sum_{j=1}^{N_{r}} \boldsymbol{P}\left( i,j | \theta\right){i^{2}j}^{2}}{N_{z}(\theta)}$$

5. LongRunEmphasis (LRE):

$$LRE=\frac{\sum_{i=1}^{N_{g}} \sum_{j=1}^{N_{r}} \boldsymbol{P}\left( i,j | \theta\right)j^{2}}{N_{z}(\theta)}$$

6. HighGrayLevelRunEmpha (HGLRE):

$$HGLRE=\frac{\sum_{i=1}^{N_{g}} \sum_{j=1}^{N_{r}} \boldsymbol{P}\left( i,j | \theta\right)i^{2}}{N_{z}(\theta)}$$

7.RunPercentage (RP):

$$RP=\frac{N_{z}(\theta)}{N_{p}}$$

8.RunLengthNonuniformity (RLN):

$$RLN=\frac{\sum_{j=1}^{N_{r}} ({\sum_{i=1}^{N_{g}} \boldsymbol{P}\left( i,j | \theta\right))}^{2}}{N_{z}(\theta)}$$

9.ShortRunEmphasis (SRE):

$$SRE=\frac{\sum_{i=1}^{N_{g}} \sum_{j=1}^{N_{r}} \frac{\mathbf{P}\left( i,j | \theta\right)}{j^{2}}}{N_{z}(\theta)}$$

10.ShortRunLowGrayLevelEmpha (SRLGLE):

$$SRLGLE=\frac{\sum_{i=1}^{N_{g}} \sum_{j=1}^{N_{r}} \frac{\mathbf{P}\left( i,j | \theta\right)}{i^{2}j^{2}}}{N_{z}(\theta)}$$

11. ShortRunHighGrayLevelEmpha (SRHGLE):

$$SRHGLE=\frac{\sum_{i=1}^{N_{g}} \sum_{j=1}^{N_{r}} \frac{\mathbf{P}\left( i,j | \theta\right)i^{2}}{j^{2}}}{N_{z}(\theta)}$$

Group 3. Shape and size based features

This group of features we include descriptors of the 3D size and shape of the ROI.

Let:

- *V* be volume of ROI.
- *A* be the surface area of the volume of interest.

1. Compactness1:

1. Compactness2:
2. Convex: Measure the proportion of the pixels in the convex hull that are also in the region
3. SurfaceArea: The surface area of the binary mask and defined as:

$$A=\sum_{i=1}^{N} \frac{1}{2}\left| \mathbf{a}_{i}\mathbf{b}_{i}ⅹ\mathbf{a}_{i}\mathbf{c}_{i} \right|$$

Where *N* is the total number of triangles covering the surface and **a**, **b** and  **c are edge vectors of the triangles.**

5.Volume: The physical volume treating the edge voxels differently.

6.Sphericity:

7. Max3DDiameter: Largest pairwise Euclidean distance between voxels on the surface of the tumor volume.
